# Discovery of a natural small-molecule compound that suppresses tumor EMT, stemness and metastasis by inhibiting TGFβ/BMP signaling in triple-negative breast cancer

**DOI:** 10.1186/s13046-019-1130-2

**Published:** 2019-03-21

**Authors:** Lei Di, Li-Juan Liu, Yong-Ming Yan, Rong Fu, Yi Li, Ying Xu, Yong-Xian Cheng, Zhao-Qiu Wu

**Affiliations:** 1 State Key Laboratory of Natural Medicines, Jiangsu Key Laboratory of Carcinogenesis and Intervention, School of Basic Medicine and Clinical Pharmacy, China Pharmaceutical University, Nanjing 211198, China; Collaborative Innovation Center for Gannan Oil-Tea Camellia Industrial Development, Gannan Medical University, Ganzhou, China; 2 Guangdong Key Laboratory for Genome Stability & Disease Prevention, School of Pharmaceutical Sciences, Shenzhen University Health Science Center, 3688 Nanhai Ave, Shenzhen, 518060 China

**Keywords:** ZL170, Triple-negative breast cancer, Epithelial–mesenchymal transition, TGFβ/BMP, Metastasis

## Abstract

**Background:**

The transforming growth factor β (TGFβ) and bone morphogenetic protein (BMP) signaling pathways are both constitutively activated in triple-negative breast cancer (TNBC). We are interested in isolating the naturally-derived small-molecule inhibitor that could simultaneously targeting TGFβ/BMP pathways and further studying its anti-proliferative/−metastatic effects as well as the underlying mechanisms in multiple tumor models.

**Methods:**

Multiple in vitro cell-based assays are used to examine the compound’s inhibitory efficacy on TNBC cell growth, stemness, epithelial-mesenchymal transition (EMT), invasion and migration by targeting TGFβ/BMP signaling pathways. Transgenic breast cancer mouse model (*MMTV-PyMT*), subcutaneous xenograft and bone metastasis models are used to examine ZL170’s effects on TNBC growth and metastasis potentials in vivo.

**Results:**

ZL170 dose-dependently inhibits cell proliferation, EMT, stemness, invasion and migration in vitro via specifically targeting canonical TGFβ/BMP-SMADs pathways in TNBC cells. The compound significantly hinders osteolytic bone metastasis and xenograft tumor growth without inflicting toxicity on vital organs of tumor-bearing nude mice. ZL170 strongly inhibits primary tumor growth and lung metastases in *MMTV-PyMT* transgenic mice. ZL170-treated tumors exhibit impaired TGFβ/BMP signaling pathways in both epithelial and stromal compartments, thereby creating a suppressive tumor microenvironment characterized by reduced extracellular matrix deposition and decreased infiltration of stromal cells.

**Conclusions:**

ZL170 inhibits tumor EMT, stemness and metastasis and could be further developed as a potent anti-metastatic agent used in combination with cytotoxic drugs for treatment of TNBC and other advanced metastatic cancers.

**Electronic supplementary material:**

The online version of this article (10.1186/s13046-019-1130-2) contains supplementary material, which is available to authorized users.

## Background

The transforming growth factor β (TGFβ) superfamily of cytokines, which comprises three TGFβ isoforms (TGFβ1, TGFβ2 and TGFβ3), bone morphogenetic proteins (BMPs; BMP1 ~ BMP7), nodals, activins, inhibins and others, is evolutionarily conserved and actively involved in many cellular processes including cell proliferation, migration, invasion, epithelial-mesenchymal transition (EMT), extracellular matrix (ECM) remodeling, angiogenesis and immune suppression [1–4]. TGFβ and BMPs transduce their signals through two highly conserved single transmembrane serine/threonine kinase receptors, termed type II receptors (TGFBR2 for TGFβ and BMPR2 for BMPs) and type I receptors (TGFBR1 (i.e. ALK5) for TGFβ and BMPR1A/1B (i.e. ALK3/6) for BMPs). Upon ligand binding and resultant heterotetrameric receptor complex formation, the activated type II receptors phosphorylate (activate) type I receptors [1–5]. Activated TGFBR1 in turn phosphorylates both Smad2/3 and Smad1/5 (but prefers to target Smad2/3), while activated BMPR1A/1B phosphorylate Smad1/5 [1–5]. The phospho-Smad1/5 and phospho-Smad2/3 interact with common mediator Smad (co-Smad), Smad4, translocate to the nucleus and initiate transcriptional activation or transcriptional repression of target genes [1–5]. The TGFβ and BMP signaling pathways are known to play pleiotropic roles in cancer progression and their action is highly cellular context-dependent [2, 6, 7]. In tumor initiation, TGFβ and BMPs inhibit cell growth and act as tumor suppressors, while in later stages, they promote cell proliferation, EMT, stemness, invasion and metastasis [2, 6, 7].

Breast cancer is the most common malignancy and the second leading cause of cancer mortality among women in the worldwide. Among the four molecular subtypes (luminal A, luminal B, Her2-enriched and triple-negative/basal-like), triple-negative breast cancer (TNBC) displays stronger invasiveness, higher risk of metastasis and poorer prognosis [8, 9]. The existing endocrine therapy, Her2-targeted therapy and chemotherapy have been proven ineffective for TNBC treatment. TGFβ and BMP ligands as well as their receptors are chronically overexpressed in TNBC and the elevated ligand/receptor levels in tumor tissues or in patients’ plasma correlate with more metastatic phenotypes and shorter patient survivals [1, 10, 11]. Accordingly, blockade of TGFβ and BMP signaling pathways is an attractive anti-cancer therapeutic approach. A variety of small-molecule TGFβ-specific or BMP-specific inhibitors are currently in the process of development [1, 12, 13]. However, the dual inhibitors that target TGFβ and BMP kinase receptors have not been characterized and studied yet.

The potential of naturally-derived small molecule compounds as the candidates for drug discovery has been well recognized [14]. The crude extracts of *Periplaneta americana* have been used as an anti-bacterial, antiviral, anti-inflammatory, anti-tumor, anti-fibrosis, and tissue-repair agent in traditional Chinese medicine for years. We are interested in compounds thereof responsible for anticancer effects which so far remains largely unknown. In the present study, we have isolated a structurally novel small-molecule oxindole compound, ZL170 from the dry whole bodies of *P. americana*, and further identified the compound as a potent dual inhibitor of TGFβ and BMP kinase receptors. Our results indicate, for the first time, that simultaneous abrogation of TGFβ and BMP signaling pathways by systemic administration of the naturally derived TGFβ/BMP dual inhibitor could result in substantially reduced tumor growth, invasion and metastasis in TNBC and other metastatic cancer types. ZL170 could be further developed as a potent anti-metastasis agent used in combination with immune checkpoint inhibitors or cytotoxic drugs for treatment of TNBC and other advanced metastatic cancers.

## Materials and methods

### Extraction, total synthesis and characterization of ZL170

For extraction of ZL170, the dry whole bodies of *P. americana* (30 kg) were extracted by refluxing with 70% EtOH (3 × 120 L × 2 h) to give a crude extract, which was suspended in water followed by extraction with EtOAc to afford an EtOAc soluble extract (230 g). Detailed protocols are described in Supplementary information.

### Cell culture

MDA-MB-231, 4 T1 and HEK293T cells were obtained from ATCC, and MDA-MB-231-SCP2 cells were kindly provided by J. Massague (Memorial Sloan-Kettering Cancer Center, New York, USA). The cells were grown in DMEM medium (Thermo Fisher) supplemented with 10% fetal bovine serum (FBS) and 1% penicillin/streptomycin (Thermo Fisher). PyMT breast cancer cell line was generated in our laboratory [15] and cultured in DMEM/F12 medium containing 5% FBS, 10 ng/ml EGF, 500 ng/ml hydrocortisone, 5 mg/ml insulin, 20 ng/ml cholera toxin and 1% penicillin/streptomycin. Cells were tested for mycoplasma contamination every 2 weeks, and only mycoplasma-negative cells were used. All cell lines in this study were authenticated in our laboratory.

### Cell transfection

Cells were transfected using Lipofectamine2000 (Thermo Fisher) according to the manufacturer’s instructions. The luciferase activity was determined by the Dual-Luciferase Reporter Assay system kit (Promega) according to the manufacturer’s instructions.

### Cloning, virus production and infection

pGL3-SBE4, pGL3-BRE4, pLenti-HA-TGFBR1-T204D, pLenti-HA-BMPR1A-Q233D, pLKO.1-BMPR1A-shRNA and pLKO.1-TGFBR1-shRNA were generated by GenScript Biotech Inc. (Nanjing, China). To produce lentivirus, 293 T cells were transfected with transfer plasmid, psPAX2 and pMD2.G. Cells were fed with fresh medium 24 h post transfection, and conditioned medium containing viral particles was harvested 48 h and 72 h post transfection. For virus infection, target cells were incubated with a mixture of virus-containing medium and culture medium at a ratio of 1:1 for 24 h in the presence of 8 μg/ml Polybrene (Sigma). Cells were re-infected for another 24 h, recovered in fresh medium for 24 h and selected in culture medium containing puromycin for 1 week.

### Cell migration and invasion assays

For migration and invasion assays, cells were seeded in upper insert in serum free medium in the absence (for cell migration assay) or presence (for cell invasion assay) of Matrigel pre-coated on the bottom (BD Bioscience). The lower chamber was filled with complete medium. After incubation period, cells were fixed with methanol for 10 min, stained by 0.5% crystal violet and counted under microscope.

### Western blotting and antibodies

Cells were washed in pre-cold PBS and lysed using radio-immunoprecipitation assay (RIPA) buffer (Thermo Fisher) supplemented with proteinase and phosphatase inhibitors (Thermo Fisher). Cell lysates was subjected Western blotting assays as described in Supplementary information.

### RNA preparation and qRT-PCR

The total RNA was isolated with a TRIzol kit (Invitrogen). Reverse transcription of purified RNA was performed using Prime Script™ RT reagent Kit with gDNA Eraser (TaKaRa). qRT-PCR was performed using qPCR Master Mix (Vazyme). GAPDH mRNA level was recognized as an endogenous control for each target gene. Primer sequences are available upon request.

### Flow cytometry analysis

The percentage of apoptotic cells was determined by the FITC Annexin V Apoptosis Detection Kit I (BD Bioscience) according to the manufacturer’s instructions. In some experiments, ALDH activity of MDA-MB-231 cells was measured using a kit from STEMCELL Technologies according to the manufacturer’s instructions.

### RNA sequencing

The total RNA was isolated with a TRIzol kit (Invitrogen). Sequencing libraries were generated using NEBNext UltraTM RNA Library Prep Kit for Illumina (NEB) following the manufacturer’s recommendations and index codes were added to attribute sequences to each sample.

### In vivo tumor growth and bone metastasis assays

Six-week-old female athymic Balb/c *nu*/*nu* mice and MMTV-*PyMT* transgenic mice were purchased from Qinglongshan Animal Facility (Nanjing, China) and Jackson Laboratory, respectively. All mice were housed under standard specific-pathogen-free (SPF) conditions and all research involving animals strictly complied with protocols approved by the Animal Welfare and Ethics Committee of China Pharmaceutical University.

### Histological, immunofluorescent and immunohistochemical analyses

Femurs and tibias were excised, fixed in 10% neutral-buffered formalin, decalcified and embedded in paraffin. Non-skeletal organs were dissected, fixed in 3.7% formaldehyde and embedded in paraffin. Tissues were sectioned and stained. Detailed protocols are described in supplementary information.

### Micro-computed tomography (μCT) assay

Femurs and tibias were scanned using the Hiscan M1001 μCT (Hiscan Information Technology Inc., China). The X-Ray tube settings were 60 kV and 133 μA and images were acquired at 50 μm resolution. A 0.5° rotation step through a 360° angular range with 50 ms exposure per step was used. The images were reconstructed with Hiscan Reconstruct software (Hiscan Information Technology Inc., China) and analyzed with Hiscan Analyzer software (Hiscan Information Technology Inc., China). After processing with a 3-D Gaussian filter to reduce noise.

### Molecular docking

The crystal structures for docking were downloaded from Protein Data Bank (PDB). All the simulations were applied through Schrödinger. Preparation of the crystal structures of TGFβ/BMP receptors were carried out using the Protein Preparation Wizard module. Proper preparation of the ligands was accomplished by the LigPrep module. All other parameters were set to the default values. The cavity that surrounded within 10 Å of the ligand in each complex was defined as the binding site. Compounds ZL170 was docked into the binding pockets of TGFβ/BMP receptors by Glide_SP module. Then the visualization was performed by the program Pymol.

### Statistics analysis

Data were presented as mean ± SD. Statistical analysis was performed as described in each figure legend, and sample sizes were shown in each corresponding figure legend. *P* < 0.05 is considered as significant.

## Results

### The chemical structure, total synthesis and acute toxicity of ZL170

**Fig. 1:**
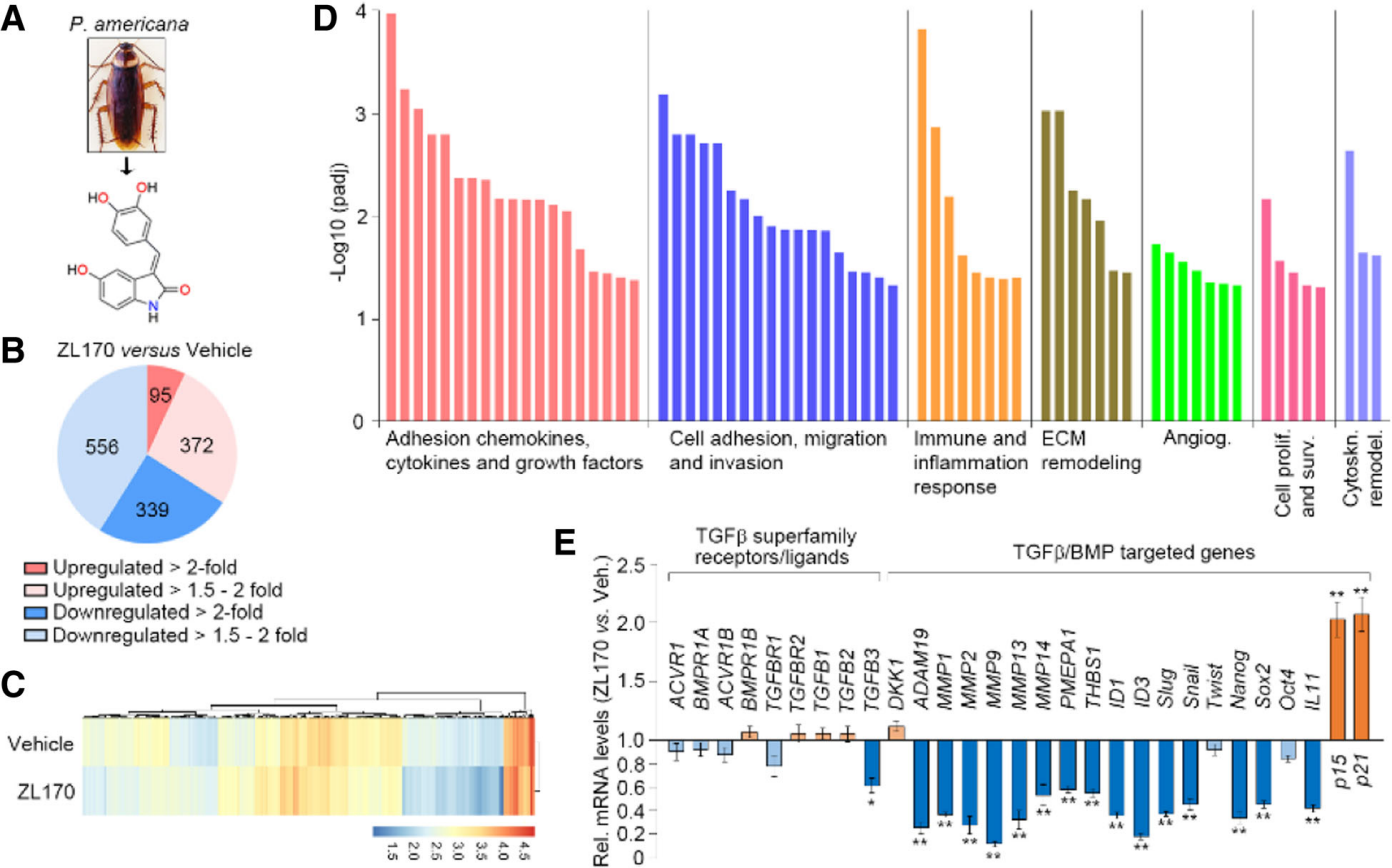
ZL170 targets the TGFβ and BMP signaling pathways in MDA-MB-231 cells. **a** Chemical structure of ZL170 that was extracted from the dry whole bodies of *P. americana*. **b-d** RNA sequencing analysis of vehicle- and ZL170-treated MDA-MB-231 cells. Pie chart denotes the distribution of total transcripts changed in ZL170-treated cells as compared with vehicle-treated cells (**b**). Heat map of all targets in vehicle- and ZL170-treated cells (**c**). Gene set enrichment analysis (GSEA) of expression of signature genes involved in multiple cellular functions (**d**). **e** qPCR analysis validated TGFβ/BMP-targeted genes as well as genes encoding TGFβ superfamily receptors and ligands. Data are represented as mean ± S.D. (*n* = 3 independent experiments). * *P* < 0.05, ** *P* < 0.01, two-sided Student’s *t*-test

ZL170 extracted from the dry whole bodies of *P. americana* is a HPLC-grade pure small-molecule oxindole compound, (E)-3-(3,4-dihydroxybenzylidene)-5-hydroxyindolin-2-one (C_15_H_11_NO_4_; MW, 269) (Fig. 1a; Additional file 1: Figure S1A). Total synthesis of ZL170 is shown in Additional file 1: Figure S1B. The detailed characterization and purity of the naturally extracted and total synthetic ZL170 are presented in Additional file 1: Figure S1C and D. Acute toxicity studies revealed that C57BL6 mice intraperitoneally (i.p.) injected with a single dose of ZL170 up to 600 mg/kg experienced no obvious adverse health effects during a 7-day observation (Additional file 1: Figure S2A).

### ZL170 specifically targets the TGFβ and BMP signaling pathways in TNBC cells

**Fig. 2:**
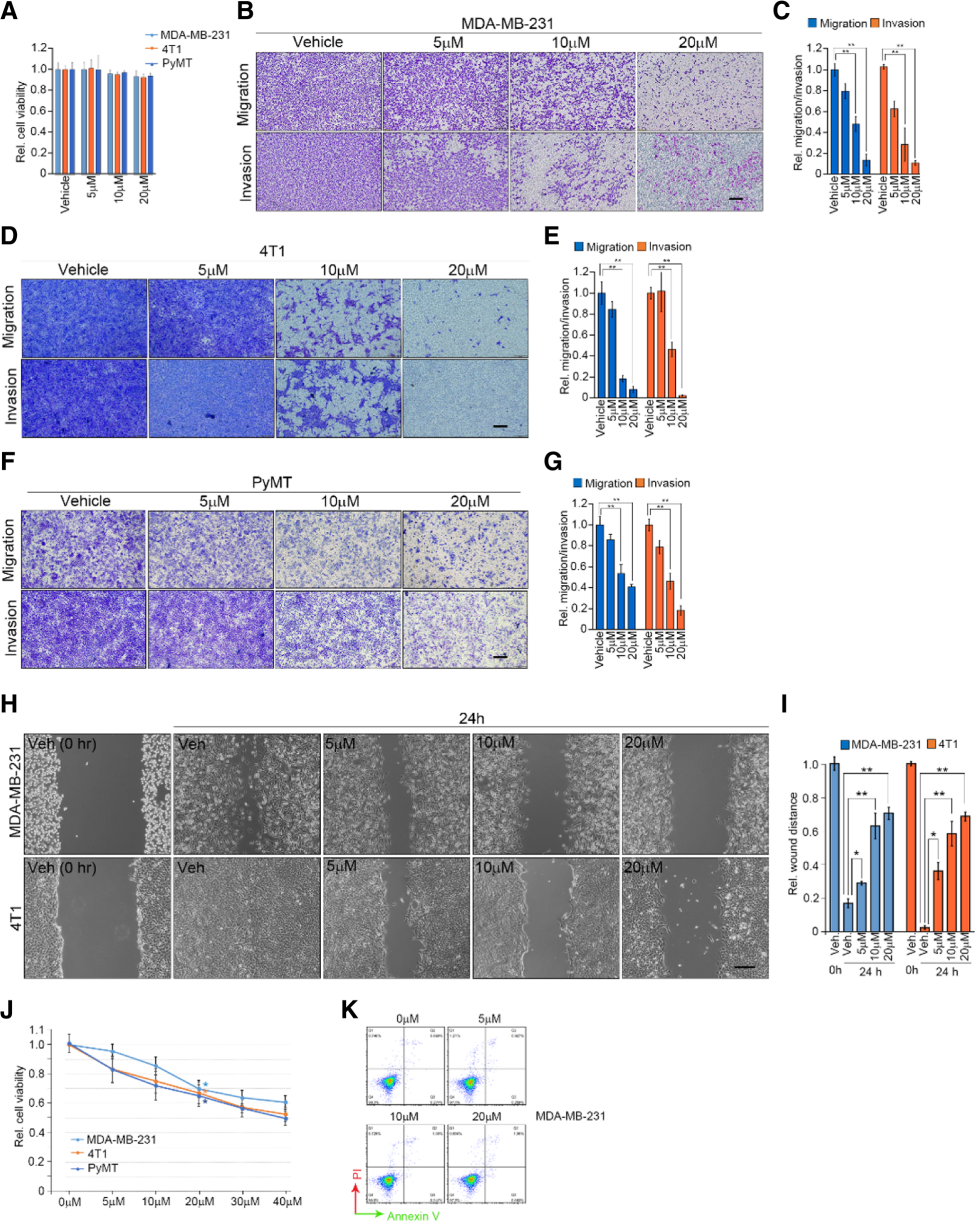
ZL170 reduces TNBC cell migration, invasion and proliferation, but does not induce apoptosis in vitro. **a** Cell viability of MDA-MB-231, 4 T1 and *PyMT* cells treated with vehicle or 20 μM ZL170 for 24 h (*n* = 3) (**b**, **d** and **f**) Boyden chamber migration and invasion assays of MDA-MB-231 (**b**), 4 T1 (**d**) and PyMT (**f**) cells (*n* = 3). Cells pretreated with ZL170 at different doses for 24 h were seeded in upper insert in the presence (for invasion assay) or absence (for migration assay) of pre-coated Matrigel. **c**, **e** and **g** Quantification of the migrated or invaded cells as shown in **c**, **e** and **g**, respectively. **h** Migration wound healing assay of MDA-MB-231 and 4 T1 cells in the presence of different doses of ZL170. **i** Quantification of the wound healing as shown in **h**. **j** Cell viability of MDA-MB-231, 4 T1 and *PyMT* cells treated with vehicle or different doses of ZL170 for 48 h (*n* = 3). **k** Cell apoptosis analysis of MDA-MB-231 cells that were treated with vehicle or different doses of ZL170 for 48 h (*n* = 3). Cells were co-stained with Annexin V and PI. Data are represented as mean ± S.D. * *P* < 0.05, ** *P* < 0.01, one-way ANOVA test. Scale bars, 200 μm (**b**, **d**, **f**, **h**)

We first sought to determine whether ZL170 could affect cell migration and invasion in a variety of basal, TNBC cell lines. As shown in Fig. 2a-i, without affecting cell viability, ZL170 dose-dependently inhibited cell migration and invasion in vitro in two well-established TNBC cell lines (MDA-MB-231 and 4 T1) as well as in a primary cell line derived from late-stage MMTV-PyMT breast tumors (TNBC-like cells; [15]). After 48 h treatment, ZL170 inhibited cell proliferation in a dose-dependent manner (by ~ 35% at 20 μM) but did not induce obvious apoptosis in the TNBC cell lines (Fig. 2j and k). To assess the global impact of ZL170 on TNBC cell function in an unbiased fashion, mRNA was isolated from vehicle- and ZL170-treated MDA-MB-231 cells and subjected to RNA sequencing. Using a minimum of two-fold change as a cutoff, ZL170 altered the expression of 434 unique transcripts (95 transcripts increased and 339 transcripts decreased; Fig. 1b and c). Gene set enrichment analysis (GSEA) further revealed that ZL170 modulated the expression of signature genes involved in regulating cell adhesion, migration, invasion, metastasis and proliferation, extracellular matrix (ECM) remodeling, cytoskeleton remodeling, immune and inflammatory response as well as angiogenesis (Fig. 1d). Notably, ZL170-treated cells demonstrated reduced expression of ECM remodeling genes (*MMP1*, *MMP2*, *MMP9*, *MMP13*, *MMP14*), bone metastasis-promoting genes (*ADAM19*, *PMEPA1*, *THBS1*, *IL11*), cell growth-stimulating genes (*ID1* and *ID3*), EMT-TFs (*Snail* and *Slug*) and CSC-TFs (*SOX2* and *Nanog*) but increased expression of tumor suppressors (*p15* and *p21*), all of which are recognized as direct TGFβ and BMP targets [2, 7, 16, 17] and were further confirmed by reverse transcription-quantitative PCR (RT-qPCR) (Fig. 1c and e). We next assessed whether ZL170 could affect other signaling pathways that are actively involved in tumor growth, invasion and metastasis. Interestingly, we found that ZL170 had no effect on p38 MAPK, ERK, JNK, PI3K-AKT, NF-κB or Notch pathways (Additional file 1: Figure S3A), suggesting that ZL170 specifically targets TGFβ and BMP signaling pathways in TNBC cells.

### ZL170 inhibits kinase activities of TGFβ and BMP receptors and impairs activation of Smads in TNBC cells

**Fig. 3:**
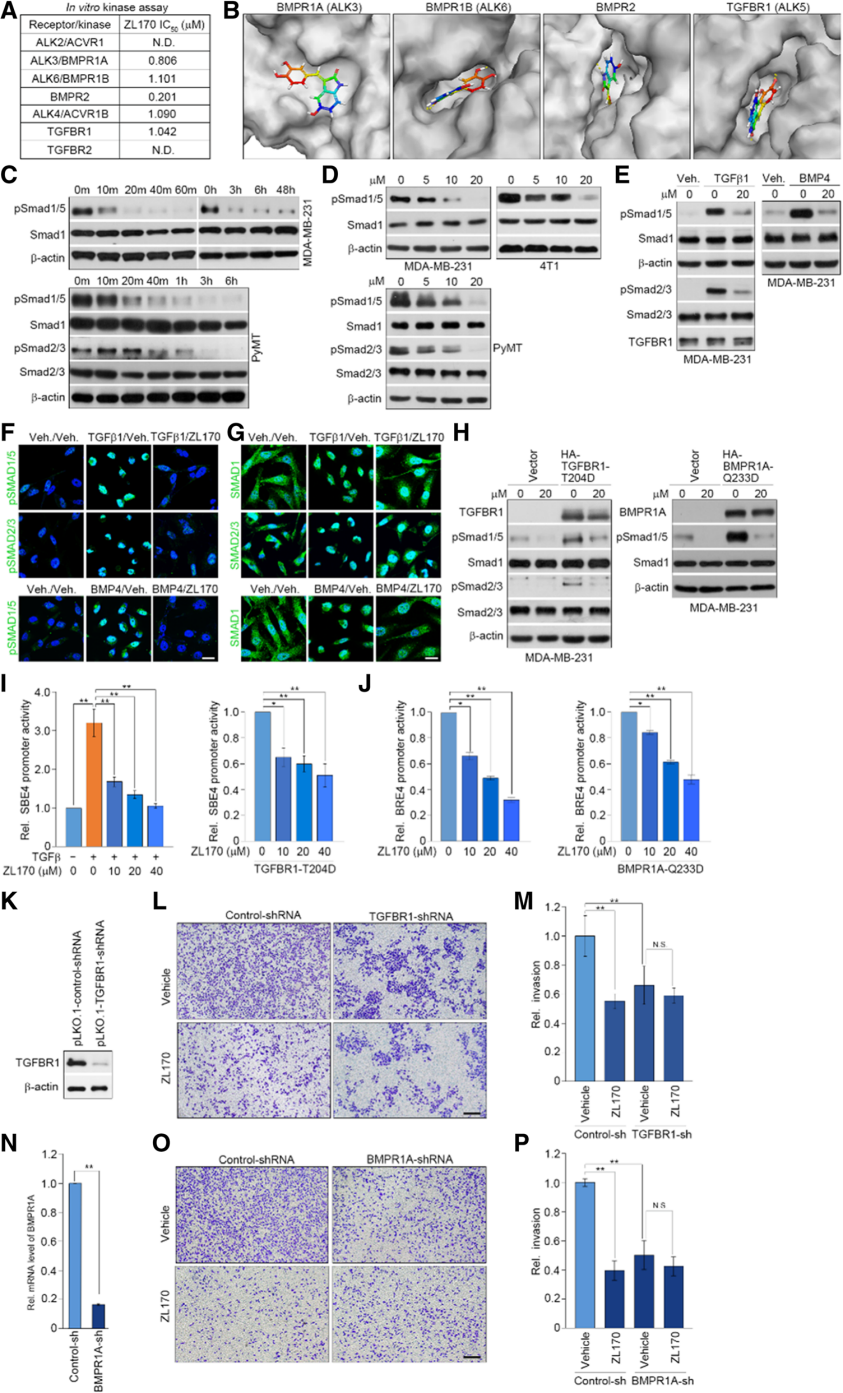
ZL170 is a dual inhibitor of TGFβ and BMP kinase receptors and reduces activation of Smads in TNBC cells. **a** In vitro kinase activity assays of the inhibitory efficacy of ZL170 on phosphorylation of the substrates (BMPR1A, BMPR1B, BMPR2, TGFBR1, ACVR1B, ACVR1 and TGFBR2). **b** Molecular docking analysis of the potential binding between ZL170 and TGFβ/BMP receptors. Illustration of surface crystal structure of ZL170 against BMPR1A, BMPR1B, BMPR2 and TGFBR1 shown. **c** Representative immunoblot analyses of the levels of phospho-Smad1/5 and phospho-Smad2/3 (and their total forms) in MDA-MB-231 and *PyMT* cells that were treated with vehicle or ZL170 at 20 μM for different times (*n* = 3). **d** Immunoblot analyses of the levels of phospho-Smad1/5 and phospho-Smad2/3 (and their total forms) in the indicated cells that were treated with vehicle or ZL170 at 5, 10 and 20 μM for 3 h. *n* = 3 independent experiments. **e** ZL170 efficiently abolished TGFβ1-stimulated and BMP4-stimulated expression of phospho-Smads in MDA-MB-231 cells as indicated by representative immunoblot analyses (*n* = 3). Cells were treated with TGFβ1 or BMP4 in combination with ZL170 at 20 μM for 3 h. **f** and ***g*** Representative immunofluorescent staining of phospho-Smads (**f**) and Smads (**g**) in resting, TGFβ1-stimulated and BMP4-stimulated MDA-MB-231 cells (*n* = 3). (*H*) ZL170 efficiently reduced the levels of phospho-Smads in TGFBR1-T204D (*left panel*) or BMPR1A-Q233D (*right panel*) stably expressing cells as indicated by representative immunoblot analyses (*n* = 3). Cells were treated with the compound for 3 h. **i** SBE promoter luciferase reporter assays in MDA-MB-231 cells that were treated with TGFβ1 and increasing doses of ZL170 (*left panel*) or in TGFBR1-T204D stably expressing cells treated with increasing doses of ZL170 (*right panel*) (*n* = 3). **j** BRE4 promoter luciferase reporter assays in MDA-MB-231 cells treated with increasing doses of ZL170 (*left panel*) or in BMPR1A-Q233D stably expressing cells treated with increasing doses of ZL170 (*right panel*). **k***-***m** Boyden chamber invasion assays of MDA-MB-231 cells stably expressing control-shRNA or TGFBR1-shRNA (**k** and **l**). Quantification of invaded cells were shown in (*M*). *n* = 3 independent experiments. **n***-***p** Boyden chamber invasion assays of MDA-MB-231 cells stably expressing control-shRNA or BMPR1A-shRNA (**n** and **o**). Quantification of invaded cells were shown in (**p**). *n* = 3 independent experiments. Data are represented as mean ± S.D. * *P* < 0.05, ** *P* < 0.01, one-way ANOVA test. Scale bars = 20 μm (**f**, **g**) and 200 μm (**l**, **o**)

Because ZL170 specifically targets the TGFβ and BMP signaling pathways, we first tested whether the compound could directly affect kinase activities of TGFβ and BMP receptors. In vitro kinase activity assay revealed that ZL170 inhibited enzymic activities of BMPR1A, BMPR1B, BMPR2 and TGFBR1 with IC_50_ values of 0.806, 1.101, 0.201 and 1.042 μM, respectively (Fig. 3a). These results suggest that ZL170 is a dual inhibitor of TGFβ and BMP kinase receptors with higher activity against BMP receptors. Due to the promising selectivity profile, molecular docking was used to further illustrate the kinase activity. We compared binding models of ZL170 on TGFβ and BMP receptors. As shown, the docking results confirmed the classic binding mode of indolinones into known kinase ATP pockets (Fig. 3b; Additional file 1: Figure S3B). ZL170 was soaked into cavity of each kinase domain and lactam moiety of ZL170 could interact with His43 of BMPR1A, His282 of BMPR1B and His283 of TGFBR1, respectively, to form a conserved hydrogen. The phenolic hydroxy substituent of ZL170 occupied the gatekeeper Phe262 of TGFBR1, which contributes greatly to the strong protein occupancy. The phenolic hydroxy moiety of ZL170 developed bidentate interaction with Tyr282 of BMPR2, and a shallow hydrophobic pocket (consisting of Gly210 and Arg211) adjacent to the hinge binding area further facilitates the interaction. The lactam moiety of ZL170 is toward the hydrophobic pocket, indicating a close interaction between ZL170 and BMPR2. We next assessed the inhibitory efficacy of ZL170 on receptor kinase activity in TNBC cells where the TGFβ and BMP signaling pathways are always inappropriately upregulated [1, 10, 11]. We observed that phospho-Smad1/5 levels started to decrease at 10 min after treatment of MDA-MB-231 cells with 20 μM ZL170 (phospho-Smad2/3 was undetectable in the cells) (Fig. 3c). Similarly, ZL170 decreased the levels of both phospho-Smad1/5 and phospho-Smad2/3 in PyMT cells in a time-dependent manner (Fig. 3c). ZL170 dose-dependently reduced phospho-Smad1/5 and (or) phospho-Smad2/3 levels in MDA-MB-231, 4 T1 and PyMT cells (Fig. 3d). It has been reported that exogenously-derived TGFβ1 triggers phosphorylation of both Smad2/3 and Smad1/5 (but prefers to phosphorylate Smad2/3), while BMP4 preferably phosphorylates Smad1/5 [5]. We therefore tested whether TGFβ- and BMP-stimulated Smad phosphorylation could be attenuated by ZL170. As shown, TGFβ1-stimulated Smad2/3 and Smad1/5 phosphorylation and BMP4-stimulated Smad1/5 phosphorylation were efficiently attenuated by ZL170 treatment of TNBC cells, as assessed by immunoblot and immunofluorescence analyses (Fig. 3e and f; Additional file 1: Figure S3C). In addition, ZL170 treatment of MDA-MB-231 cells efficiently reversed TGFβ1-induced Smad1 and Smad2/3 nuclear translocation and completely abolished BMP4-induced Smad1 nuclear translocation (Fig. 3g). Expression of a constitutively active TGFBR1-T204D mutant or BMPR1A-Q233D mutant in MDA-MB-231 cells substantially increased the levels of phospho-Smad2/3 and phospho-Smad1/5, which could be almost completely abolished by ZL170 treatment (Fig. 3h). Increased SBE4 (which contains four copies of Smad binding elements) transcription that was observed in TGFβ1-stimulated cells or in TGFBR1-T204D stably expressing cells was efficiently reversed in the presence of ZL170 (Fig. 3i). ZL170 suppressed BRE4 (a BMP/SMAD transcriptional reporter) promoter activity in MDA-MB-231 cells or in BMPR1A-Q233D stably expressing cells in a dose-dependent manner (Fig. 3j). To directly test whether TGFBR1 or BMPR1A is involved in the inhibition of ZL170 on cell invasion, MDA-MB-231 cells were infected with TGFBR1- or BMPR1A-shRNA and subjected to in vitro cell invasion analysis. As shown, TGFBR1-depleted cells exhibited impaired cell invasion relative to control cells, and did not respond to ZL170 treatment (Fig. 3k-p), suggesting that TGFβR1 or BMPR1A serves as a direct target of ZL170 and is indeed involved in ZL170-mediated inhibition of cell invasion.

### ZL170 reduces snail and slug expression, suppresses the EMT program and impairs EMT-dependent formation of filopodium-like protrusions (FLPs) in TNBC cells

**Fig. 4:**
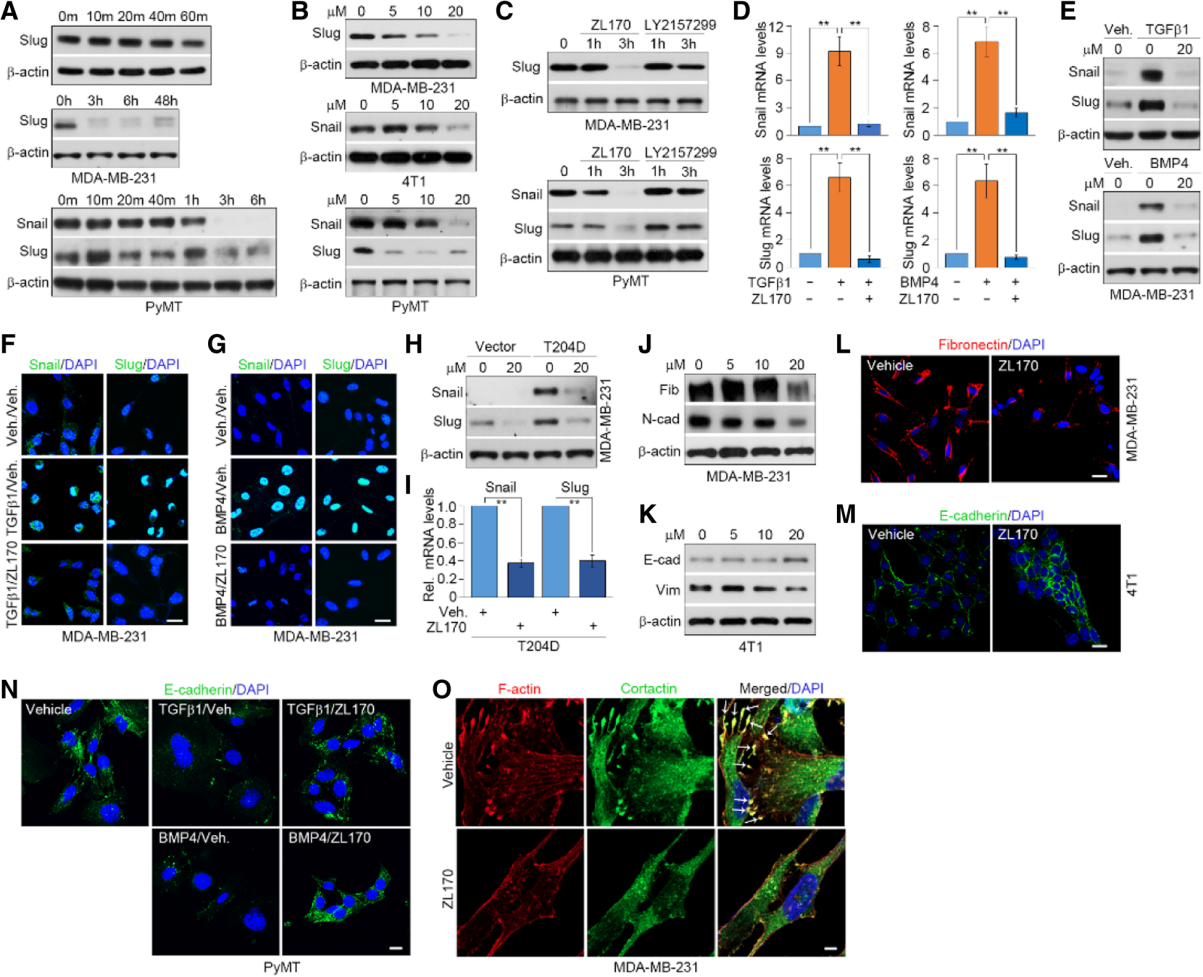
ZL170 inhibits Snail and Slug expression, reverses the EMT program and reduces EMT-dependent FLP formation in TNBC cells. **a** Representative immunoblot analyses of Snail and Slug levels in MDA-MB-231 and *PyMT* cells that were treated with vehicle or ZL170 at 20 μM for different times (*n* = 3). **b** and **c** Immunoblot analyses of Snail and Slug levels in the indicated cells that were treated with vehicle or ZL170 at 5, 10 and 20 μM for 3 h (**b**) or in the indicated cells that were treated with vehicle, ZL170 or LY2157299 at 20 μM for 1 h and 3 h (**c**). *n* = 3 independent experiments. **d-g** ZL170 efficiently abolished TGFβ1-stimulated and BMP4-stimulated expression of Snail and Slug in MDA-MB-231 cells as indicated by qPCR (**d**), immunoblot (**e**) and immunofluorescent (**f** and **g**) analyses (*n* = 3). Cells were treated with TGFβ1 or BMP4 in combination with ZL170 at 20 μM for 3 h. Data are represented as mean ± S.D. ** *P* < 0.01, one-way ANOVA test. **h** and **i** ZL170 efficiently reduced Snail and Slug levels in TGFBR1-T204D stably expressing cells as indicated by representative immunoblot (**h**) and qPCR (**i**) analyses (*n* = 3). Cells were treated with the compound for 3 h. **j** and **k** Representative immunoblot analyses of fibronectin, N-cadherin, vimentin or E-cadherin levels in MDA-MB-231 (**j**) and 4 T1 (**k**) cells that were treated with vehicle or ZL170 at 5, 10 and 20 μM for 48 h (n = 3). (*L* and *M*) Immunofluorescent staining of fibronectin (**l**) and E-cadherin (**m**) in the indicated cells that were treated as described in *J* and *K*, respectively (n = 3). **n** Immunofluorescent staining of E-cadherin in the indicated cells that were treated with TGFβ1 or BMP4 in combination with ZL170 at 20 μM for 48 h (n = 3). **o** Immunofluorescent staining of F-actin and cortactin in vehicle- and ZL170-treated MDA-MB-231 cells. Nuclear, DAPI (blue). Arrows denote F-actin^+^/cortactin^+^ FLPs (n = 3). Scale bars = 20 μm (**f**, **g**, **l**, **m**, **n**) and 5 μm (**o**)

EMT-TFs Snail and Slug have been identified as TGFβ and BMP targets [7, 17]. RNA sequencing and RT-qPCR analyses showed that mRNA levels of Snail and Slug were substantially reduced in ZL170-treated TNBC cells in which the TGFβ and BMP pathways were robustly impaired (Fig. 1c and e; Additional file 1: Figure S4A). Immunoblot analysis further revealed that protein levels of Snail and Slug started to decrease at 3 h after treatment of MDA-MB-231 and PyMT cells with 20 μM ZL170 (Fig. 4a). ZL170 dose-dependently decreased Snail and Slug protein levels in three TNBC cell lines (Fig. 4b). Interestingly, ZL170 treatment (20 μM, 3 h) efficiently reduced Snail and Slug protein levels, while treatment of LY2157299 (20 μM, 3 h), a TGFβ-specific inhibitor which is currently in phase I/II clinical trials, did not alter Snail or Slug protein expression in MDA-MB-231 and PyMT cells (Fig. 4c), suggesting that ZL170 has a stronger inhibitory effect on Snail and Slug expression than LY2157299. As expected, TGFβ1 and BMP4 potently stimulated Snail and Slug expression at both mRNA and protein levels, which was completely abolished by ZL170 (Fig. 4d-g; Additional file 1: Figure S4B and C). Expression of a constitutively active TGFBR1-T204D mutant in MDA-MB-231 cells markedly increased Snail and Slug expression levels, which was efficiently reversed in the presence of ZL170 (Fig. 4h and i). ZL170 treatment of TNBC cells increased epithelial features but reduced mesenchymal features, as evidenced by the increased levels of epithelial markers such as E-cadherin and Keratin 18 but decreased expression of mesenchymal markers such as fibronectin, N-cadherin and vimentin (Fig. 4j-m; Additional file 1: Figure S4D-F). Furthermore, TGFβ1 and BMP4 significantly decreased E-cadherin expression in PyMT cells, and the decreased E-cadherin was efficiently restored in the presence of ZL170 (Fig. 4n). It has been reported that EMT-dependent induction of tumor-initiating and metastasis-forming potentials involves and depends on the enhancement of assembled filopodium-like protrusions (FLPs), cytoskeletal actin-rich protrusions morphologically resembling filopodia formed by aggressive cancer cells [18]. These observations, together with our present demonstration that ZL170 efficiently suppressed EMT program in TNBC cells, promoted us to ask whether ZL170 could affect FLP formation in MDA-MB-231 cells. Colocalization of F-actin with the actin-bundling protein cortactin [19] was used to identify FLPs. As shown, FLPs can be readily detected on the surface of control cells, while FLPs were almost completely absent in ZL170-treated cells (Fig. 4o). Together, these findings suggest that ZL170 treatment of TNBC cells reduces EMT-TF expression, suppresses the EMT program and impairs EMT-dependent FLP formation, thereby hindering TNBC cell growth, invasion and metastasis.

### ZL170 suppresses stemness function in TNBC cells

**Fig. 5:**
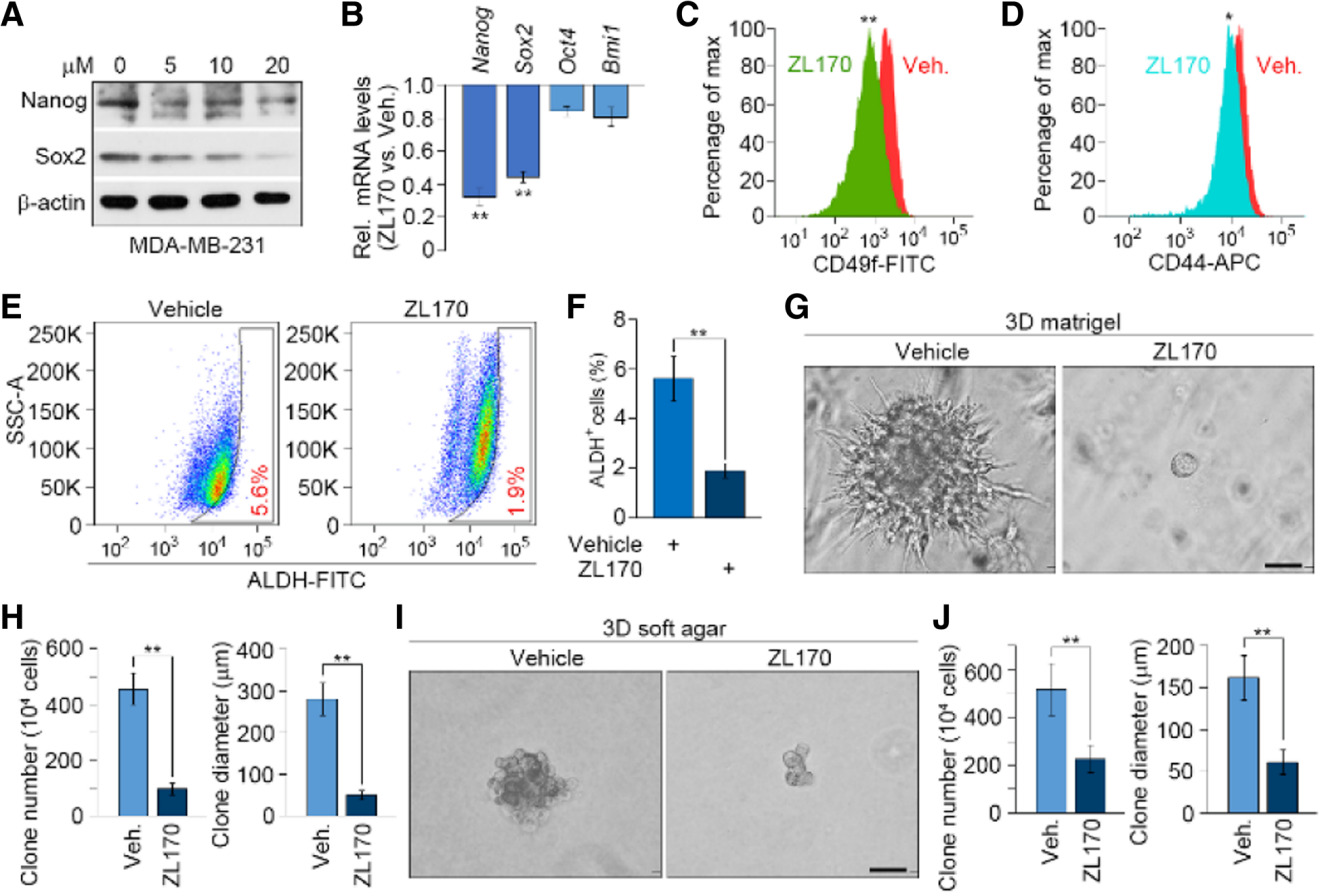
ZL170 reduces stemness function in MDA-MB-231 cells. **a** Immunoblot analyses of Nanog and Sox2 levels in MDA-MB-231 cells that were treated with vehicle or ZL170 at 5, 10 and 20 μM for 48 h (*n* = 3). **b** qPCR analysis of *Nanog*, *Sox2*, *Oct4* and *Bmi1* levels in cells that were treated as described in *A* (*n* = 3). **c** and **d** Flow cytometry analysis of CD49f (**c**) and CD44 (**d**) cell-surface expression in MDA-MB-231 cells that were treated with vehicle or ZL170 for 48 h (*n* = 3). **e** and **f** Representative histogram (**e**) and quantification (**f**) of ALDH^+^ subpopulation in MDA-MB-231 cells that were treated with vehicle or ZL170 for 48 h (*n* = 3). **g-j** Representative phase-contrast images of vehicle-treated or ZL170-treated MDA-MB-231 cells that were embedded in 3D Matrigel (**g**) or 3D soft agar gel (**i**), and tumor sphere number and diameter quantified (**h** and **j**). *n* = 3 independent experiments. Data are represented as mean ± S.D. * *P* < 0.05, ** *P* < 0.01, two-sided Student’s *t*-test. Scale bars = 50 μm (**g**, **i**)

TGFβ and BMP signaling pathways promote expression of EMT-TFs and cancer stem cell (CSC)-associated TFs (CSC-TFs) to induce EMT and increase stemness function in aggressive cancer cells, thereby facilitating tumor invasion and metastasis [1, 17]. Accordingly, we evaluated whether ZL170 could affect stemness function in TNBC cells. We found that ZL170 treatment of MDA-MB-231 cells markedly reduced Nanog and Sox2 expression at both mRNA and protein levels but did not affect Oct4 or Bmi1 expression (Fig. 1e; Fig. 5a and b). ZL170 also decreased levels of CD49f and CD44, two markers of the CSC population [20–22], as assessed by flow cytometry analysis (Fig. 5c and d). In addition, aldehyde dehydrogenase 1 (ALDH1) has been identified as a CSC marker and a predictor of poor clinical outcome [15, 23]. We observed that ZL170 remarkably reduced the percentage of ALDH1^+^ CSC subpopulation (Fig. 5e and f). Furthermore, ZL170 remarkably reduced tumorsphere- or colony-forming potential (that is, stemness) of MDA-MB-231 cells cultured in 3-dimentional (3D) Matrigel or 3D soft agar as evidenced by fewer, smaller and less invasive tumor spheres (or colonies) formed from ZL170-treated single cells (Fig. 5g-j).

### ZL170 impairs TNBC osteolytic bone metastasis and xenograft tumor growth by targeting the TGFβ-Smads and BMP-Smads signaling pathways

**Fig. 6:**
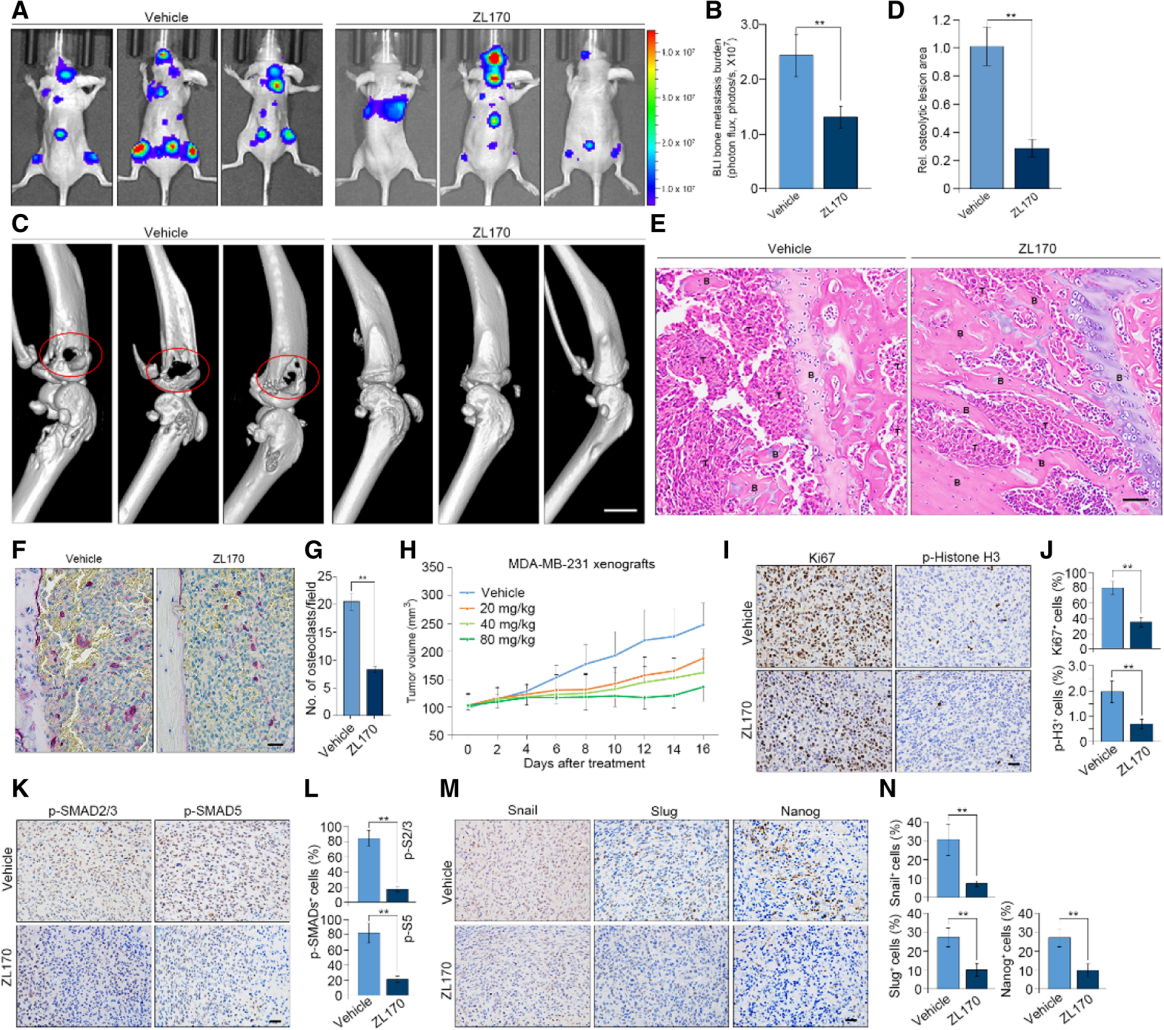
ZL170 inhibits TNBC osteolytic bone metastasis and xenograft tumor growth by targeting the TGFβ and BMP signaling pathways. **a** and **b** BLI images (**a**) and quantification (**b**) of bone lesions from nude mice that were intracardially injected with SCP2 cells labelled with firefly luciferase and then received treatment of vehicle or ZL170 (*i.p.*, 80 mg/kg/day for 2 consecutive weeks; *n* = 6 mice). **c** and **d** μ-CT images (**c**) and quantification (**d**) of osteolytic lesions from vehicle- and ZL170-treated mice (*n* = 6 mice). Circled area denotes osteolytic lesions on the bone surface and arrows depict fractured cortical bone shown in cross-section scanning images. **e** H.E. staining of bone sections from vehicle- and ZL170-treated mice (*n* = 6 mice). T, tumor cells; B, bone. **f** and **g** TRAP staining images (**f**) and quantification (**g**) of TRAP^+^ osteoclasts of bone sections from vehicle- and ZL170-treated mice. (*n* = 6). (**h**) A dose-dependent inhibition of ZL170 on the growth of MDA-MB-231 xenograft tumors (n = 6 mice). The compound was administrated at 20, 40 and 80 mg/kg for 16 consecutive days starting from day 14 when tumor volume reached ~ 100 mm^3^. **i**, **k** and **m** Immunohistochemical analyses of Ki67 and phospho-histone H3 (**i**), phospho-Smad2/3 and phospho-Smad5 (**j**), and Snail, Slug and Nanog (**i**) in vehicle- and ZL170-treated xenograft tumors (n = 6). **j**, **l** and **n** Quantification of Ki67^+^ and phospho-histone H3^+^ (**j**), phospho-Smad2/3^+^ and phospho-Smad5^+^ (**k**), and Snail^+^, Slug^+^ and Nanog^+^ (**n**) cells in the indicated xenograft tumors (*n* = 6). Data are represented as mean ± S.D. ** *P* < 0.01, two-sided Student’s *t*-test. Scale bars = 700 μm (**c**) and 20 μm (**e**, **f**, **i, k**, **m**)

The TGFβ and BMP signaling pathways are aberrantly upregulated in bone metastases samples from breast cancer patients [24]. Preclinical models have confirmed that the TGFβ and BMP signaling pathways induces the osteolytic phenotype and consequently promotes the progression of osteolytic bone metastases [3, 25]. We therefore tested the antitumor efficacy of ZL170 using a mouse model of bone metastasis. For this purpose, luciferase-labelled SCP2 cells, a highly bone metastatic MDA-MB-231 subline, were intracardially injected into the circulation of nude mice followed by bioluminescent imaging (BLI) of bone metastasis at 5 weeks post-inoculation. BLI analysis demonstrated that ZL170 (80 mg/kg/day) markedly decreased tumor bone burden 2 weeks after injection when compared to vehicle-treated mice (Fig. 6a and b). ZL170-treated mice demonstrated significantly reduced osteolytic bone lesions relative to vehicle-treated mice, as assessed by micro-computed tomography (μ-CT) and H.E. staining (Fig. 6c-e). Consistent with these results, histological analysis revealed a remarkable decrease in the numbers of tartrate-resistant acid phosphatase-positive (TRAP^+^) osteoclasts in both tibia and femur of ZL170-treated mice as compared to vehicle-treated mice (Fig. 6f and g). Overall, these data demonstrated that ZL170 significantly decreased osteolytic bone metastases of MDA-MB-231 cells. Because ZL170 significantly reduced TNBC cell growth in vitro (Fig. 2j), we next asked whether the compound could affect TNBC cell growth in vivo. Accordingly, cells were subcutaneously injected into the nude mice and ZL170 was administrated (20, 40 and 80 mg/kg/day, respectively) starting on day 14 when the xenograft tumors grew to around 100mm^3^. After 16 days of treatment, ZL170 efficiently inhibited xenograft tumor growth in a dose-dependent manner without eliciting toxicity on tumor-bearing mice (Fig. 6h; Additional file 1: Figure S5A). Consistently, a marked reduction in numbers of proliferative (Ki67^+^) and mitotic (phospho-Histone H3^+^, pH 3^+^) cells was observed in ZL170-treated tumors, whereas numbers of apoptotic (cleaved caspase 3^+^) cells were comparable between ZL170- and vehicle-treated tumors (Fig. 6i and j; Additional file 1: Figure S5B and C), confirming the in vitro results that ZL170 did not induce obvious apoptosis in cultured cells. As expected, phospho-Smad2/3 and phospho-Smad5 were substantially decreased in ZL170-treated tumors as compared to vehicle-treated tumors (Fig. 6k and l). The levels of Snail, Slug and Nanog were markedly reduced in the xenograft tumors following ZL170 treatment (Fig. 6m and n). Taken together, these results demonstrated that ZL170 efficiently reduced TNBC osteolytic bone metastasis and xenograft tumor growth by targeting TGFβ-Smad and BMP-Smad signaling pathways.

### ZL170 reduces primary tumor growth and lung metastases in PyMT transgenic mice

**Fig. 7:**
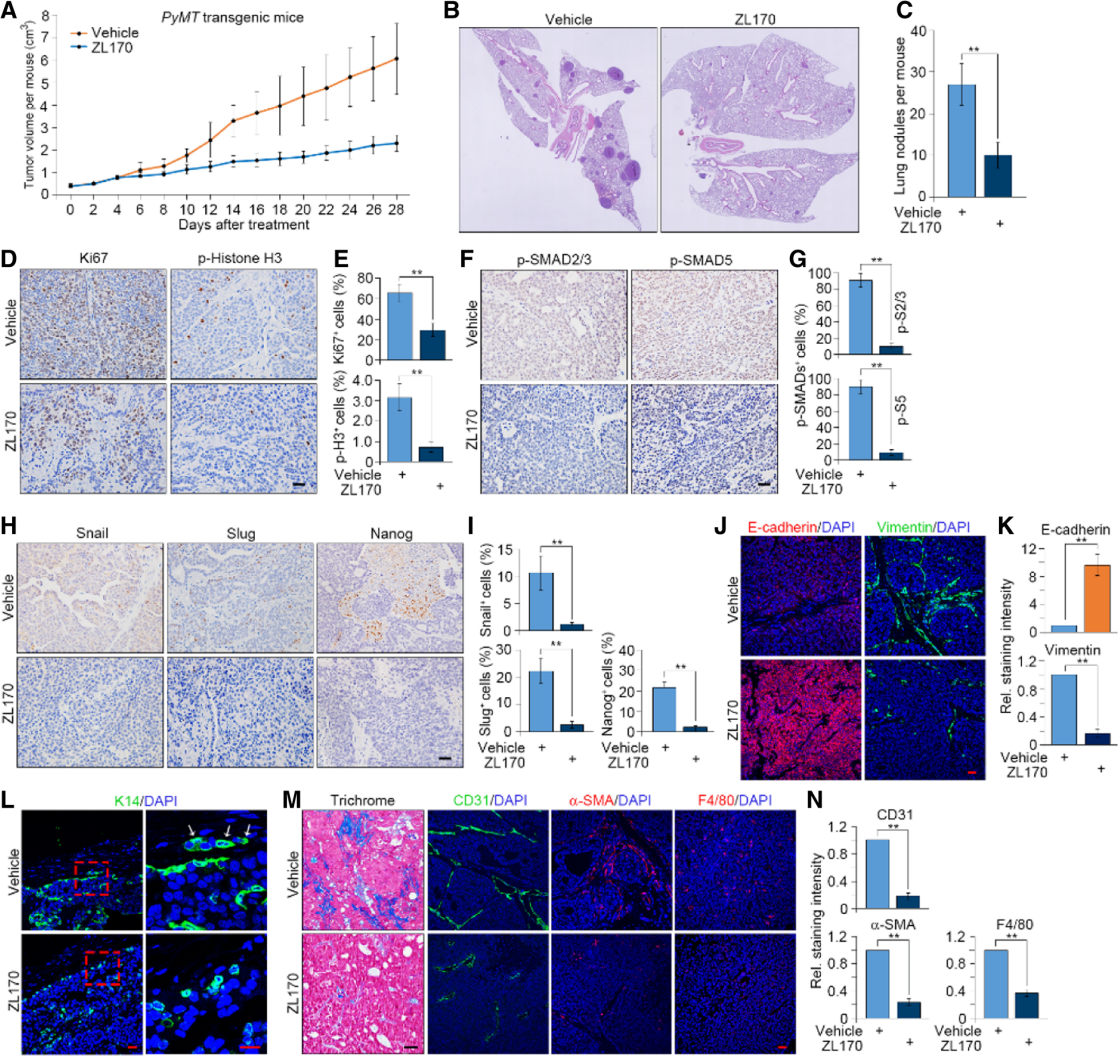
ZL170 inhibits the growth of *PyMT*-induced breast tumors and reduces their spontaneous metastases to lung. **a** Growth curves of primary breast tumors from vehicle- and ZL170-treated (*i.p.*, 80 mg/kg/day) MMTV-*PyMT* transgenic mice (*n* = 6). **b** and **c** Representative H.E. images (**b**) and quantification (**c**) of nodules in lungs from the indicated mice (*n* = 6). **d**, **f** and **h** Immunohistochemical analyses of Ki67 and phospho-histone H3 (*D*), phospho-Smad2/3 and phospho-Smad5 (**f**), and Snail, Slug and Nanog (*H*) in primary tumors of vehicle- and ZL170-treated mice (*n* = 6). **e**, **g** and **i** Quantification of Ki67^+^ and phospho-histone H3^+^ (**e**), phospho-Smad2/3^+^ and phospho-Smad5^+^ (**g**), and Snail^+^, Slug^+^ and Nanog^+^ (**i**) cells in primary tumors of the indicated mice (*n* = 6). **j***-***l** Immunofluorescent analyses of E-cadherin and vimentin (**j**) and K14 (**l**) in primary tumors of the indicated mice (*n* = 6). Quantification of staining intensity of E-cadherin and vimentin is shown (**k**). (*M*) Masson’s Trichrome staining and immunofluorescent analyses of CD31, α-SMA and F4/80 (**m**) in primary tumors of the indicated mice (*n* = 6). Nuclear, DAPI (blue). (*N*) Quantification of staining intensity of CD31, α-SMA and F4/80 as shown in **m**. Data are represented as mean ± S.D. ** *P* < 0.01, two-sided Student’s *t*-test. Scale bars = 20 μm (**d**, **f**, **h**, **j**, **l**, **m**)

We next tested whether the TGFβ/BMP dual inhibitor ZL170 could affect the progression, invasion and metastasis of spontaneous breast tumors using a MMTV-PyMT transgenic mouse model of metastatic breast cancer that mirrors the multi-step progression of human breast cancers [26]. For this purpose, 2-month-old female mice that have developed multiple spontaneous breast tumors in similar size (~ 0.4 cm^3^/mouse) were administrated with vehicle or ZL170 (40 mg/kg/day) for consecutive 4 weeks. We observed that ZL170 significantly reduced the growth of primary breast tumors without eliciting toxicity on tumor-bearing mice (Fig. 7a; Additional file 1: Figure S6A). ZL170-treated mice exhibited markedly fewer and smaller metastatic lung nodules relative to vehicle-treated mice (Fig. 7b and c). Histological analysis revealed that ZL170 remarkably decreased numbers of Ki67^+^ and pH 3^+^ cells, without increasing numbers of apoptotic cells (Fig. 7d and e; Additional file 1: Figure S6B and C). In consistent with the observations in vitro, phospho-Smad2/3 and phospho-Smad5 were markedly decreased in ZL170-treated tumors where the levels of Snail, Slug and Nanog were robustly decreased as well (Fig. 7f-i). Notably, we observed that the levels of E-cadherin were markedly increased but vimentin expression was significantly reduced in ZL170-treated tumors, suggesting that ZL170 reverses the EMT program in the in vivo set (Fig. 7j and k). Cytokeratin-14 (K14) has recently been recognized as a marker for highly migratory cancer cells in breast tumors that can initiate collective invasion, a critical step during metastatic progression of breast tumors [15, 27]. We observed that K14^+^ cells were enriched at the invasive border in vehicle-treated tumors, forming strands invading into the surrounding stromal tissues (top panel, Fig. 7l). In striking contrast, ZL170-treated tumors failed to exhibit a locally invasive phenotype and the K14-enriched invasive strands were undetectable in the stromal tissue (bottom panel, Fig. 7l). The TGFβ and BMP signaling pathways have been reported to determine microenvironmental modification that heavily influences tumor progression, invasion and metastasis [1, 3]. We therefore sought to test whether ZL170 could induce microenvironmental change in primary breast tumors. As shown, ZL170-treated tumors exhibited reduced ECM deposition and impaired infiltration of CD31^+^ endothelial cells, α-SMA^+^ cancer-associated fibroblasts as well as F4/80^+^ macrophages (Fig. 7m and n), suggesting that ZL170 treatment created a suppressive tumor microenvironment, which in turn suppressed tumor growth, invasion and metastasis in TNBC.

## Discussion

The TGFβ and BMP signaling pathways are aberrantly upregulated in various types of advanced metastatic cancers and play essential roles in controlling cancer stemness, EMT, metastasis and chemoresistance. Targeting the TGFβ and BMP signaling pathways is therefore an attractive anti-cancer therapeutic approach. Many small-molecule inhibitors that solely target TGFβ or BMP kinase receptors have been investigated for metastatic cancers in preclinical studies, and two of them are currently investigated in clinical trials [1, 12, 13, 28–30]. It should be noted that TGFβ and BMP signaling pathways are both actively involved in many cellular processes during tumor malignant progression. Targeting each pathway by the current TGFβ-specific or BMP-specific inhibitors is insufficient to block activation of Smads as well as Smad-mediated transcription of downstream targets. In support of this notion, we observe that ZL170 treatment of TNBC cell lines for 3 h almost completely abolishes expression of Snail and Slug, while treatment of LY2157299, a TGFβ-specific inhibitor does not affect Snail or Slug expression. Snail and Slug have been widely recognized as important drivers of tumor invasion and metastasis by inducing the EMT program in malignant tumor cells [31–34]. We therefore propose that the TGFβ/BMP dual inhibitor ZL170 has a stronger efficacy against TNBC growth, invasion and metastasis than the TGFβ-specific inhibitor LY2157299. Importantly, simultaneous inhibition of TGFβ and BMP signaling pathways by treatment with ZL170 might overcome chemoresistance that is frequently observed in TGFβ-specific or BMP-specific inhibitor-treated cells. However, further work is required to address this issue.

Cancer stem cells (CSCs), a minority population of the solid malignancies that possess the defining features of clonogenicity and self-renewal, are reported to be associated with tumor metastasis, recurrence and chemoresistance [15, 20, 35]. The EMT program is known to generate cancer cells with properties of stem cells characterized by increased expression of stem cell markers, augmented chemoresistance to therapy and enhanced tumor-initiating activity (that is, stemness) in vitro and in vivo [20]. CSC-TFs (e.g. Nanog and Sox2) as well as EMT-TFs (e.g. Snail and Slug) have been identified as target genes of TGFβ and BMP signaling pathways [1, 17], and most importantly, their expression levels are reduced in ZL170-treated MDA-MB-231 cultures and xenograft tumors as well as *PyMT* primary tumors, in which the EMT program is strongly suppressed as evidenced by acquisition of E-cadherin and loss of vimentin. We propose that ZL170 impairs the stemness of TNBC cells in vitro and in vivo not only by directly targeting CSC-TFs Nanog and Sox2 but also by targeting EMT-TFs Snail and Slug to inhibit EMT-conferred CSC characteristics, and thus reduces CSC-associated tumor metastasis, recurrence and chemoresistance in TNBC.

The activated TGFβ and BMP signaling pathways in epithelial and stromal compartments are both recognized to have a substantial role in regulating tumor growth, invasion and metastasis [3, 36–39]. In the present study, we find that disruption of epithelial TGFβ and BMP pathways by ZL170 may create a suppressive stromal microenvironment characterized by decreased ECM deposition as well as impaired recruitment and proliferation of cancer-associated fibroblasts (CAFs), endothelial cells and immune cells, which in turn reduces epithelial tumor growth, invasion and metastasis. On the other hand, targeting stromal TGFβ and BMP pathways by ZL170 may directly impair the recruitment, activation and function of CAFs and immune cells, which in turn reduces epithelial tumor growth, invasion and metastasis as well.

## Conclusions

The data presented here suggest that ZL170 potently inhibits tumor growth, invasion and metastasis by simultaneously targeting TGFβ and BMP signaling pathways in both epithelial and stromal compartments of TNBC tumors. The compound can be further developed as a potent anti-metastatic agent used in combination with cytotoxic drugs for treatment of TNBC as well as other advanced metastatic cancers.

## Additional file

Additional file 1: **Figure S1.** Extraction, total synthesis and characterization of ZL170. **Figure S2.** ZL170 treatment does not induce C57BL6 mice death or any obvious adverse health effects. **Figure S3.** ZL170 is a potent dual inhibitor of TGFβ and BMP kinase receptors. **Figure S4.** ZL170 reverses the EMT program in TNBC cells. **Figure S5.** Administration of ZL170 does not induce apoptosis or elicit toxicity on key organs of tumor-bearing mice. **Figure S6.** ZL170 treatment does not induce apoptosis or elicit toxicity on key organs of *PyMT* transgenic mice. (DOCX 6822 kb)

## Abbreviations

ALDH: Aldehyde dehydrogenase
ATP: Adenosine triphosphate
BFI: Biofluorescent imaging
BMP: Bone morphogenetic protein
BMPR1A: Bone morphogenetic protein receptor type 1A
BMPR1B: Bone morphogenetic protein receptor type 1B
BMPR2: Bone morphogenetic protein receptor type-2
CAF: Cancer-associated fibroblast
CSC: Cancer stem cell
ECM: Extracellular matrix
EMT: Epithelial–mesenchymal transition
EtOAc: Ethyl acetate
EtOH: Ethyl alcohol
FLP: Filopodium-like protrusion
TF: Transfer factor
TGFBR1: Transforming growth factor-beta receptor type-1
TGFβ: Transforming growth factor-beta
TNBC: Triple-negative breast cancer
TRAP: Tartrate-resistant acid phosphatase
α-SMA: α-smooth muscle actin
μ-CT: Micro-computed tomography
