# Supplementary figures and images for: Discovery of a natural small-molecule compound that suppresses tumor EMT, stemness and metastasis by inhibiting TGFβ/BMP signaling in triple-negative breast cancer
Source: J Exp Clin Cancer Res. 2019 Mar 21;38:134. doi: 10.1186/s13046-019-1130-2 (PMC6429712; doi:10.1186/s13046-019-1130-2)

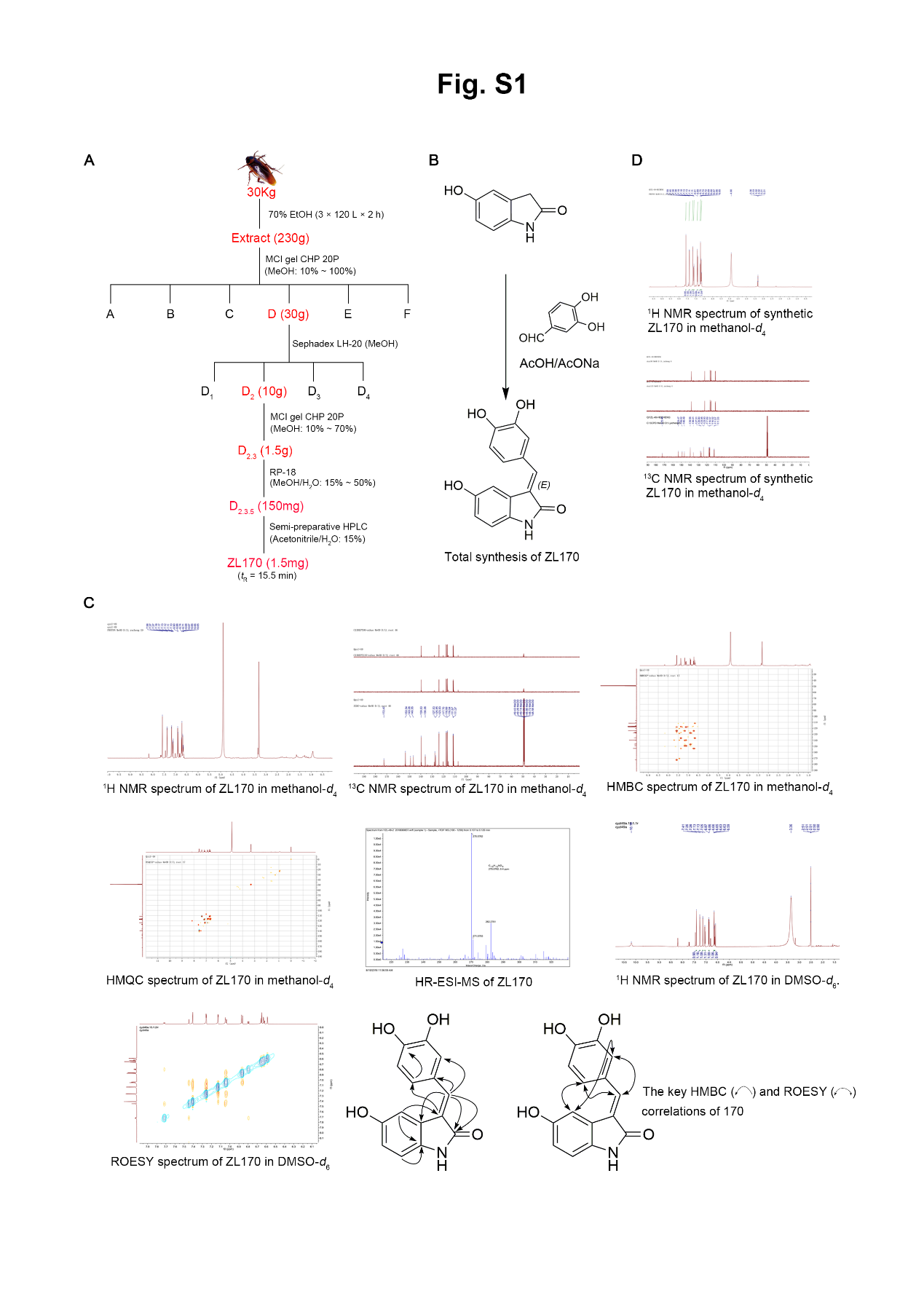

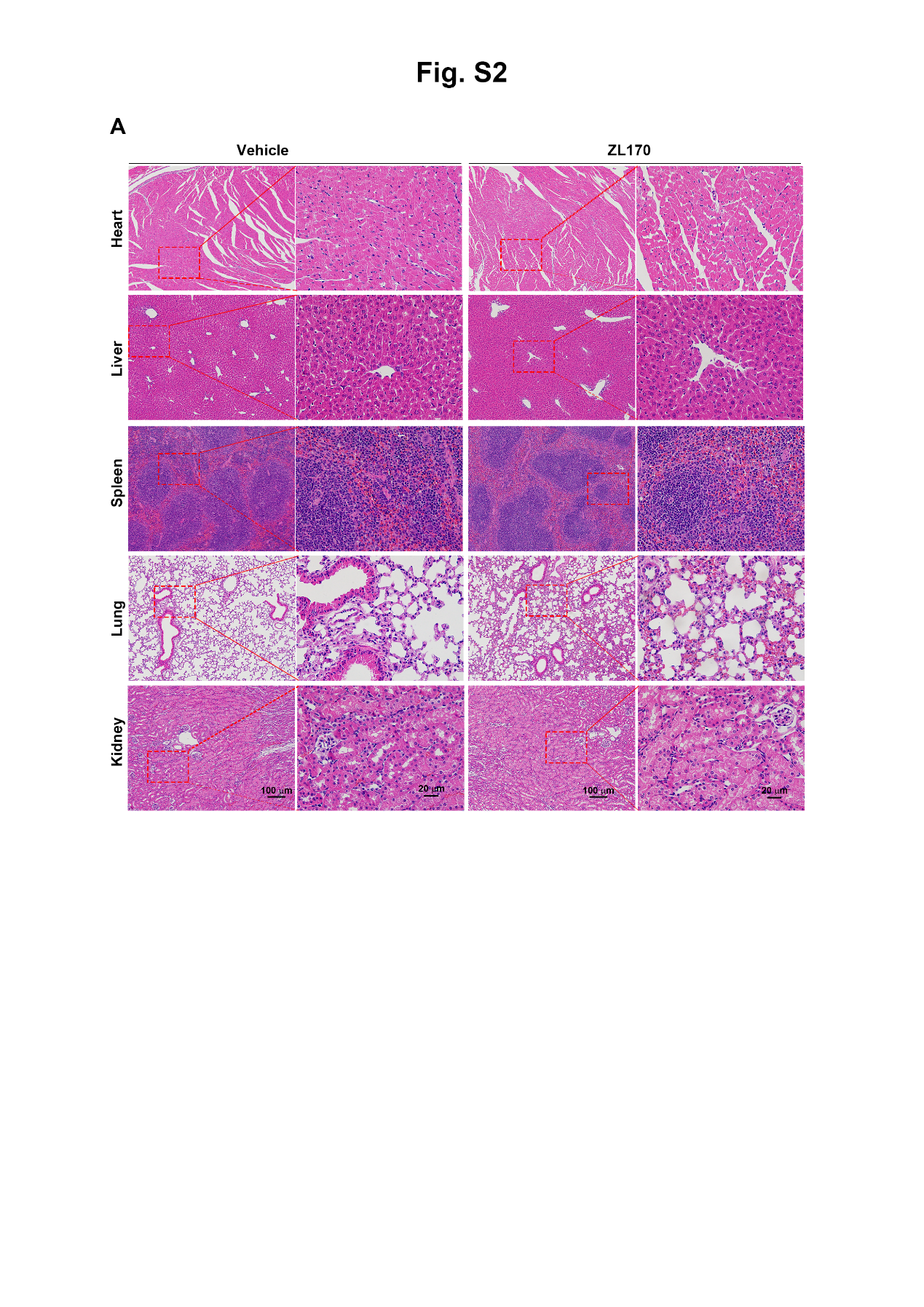

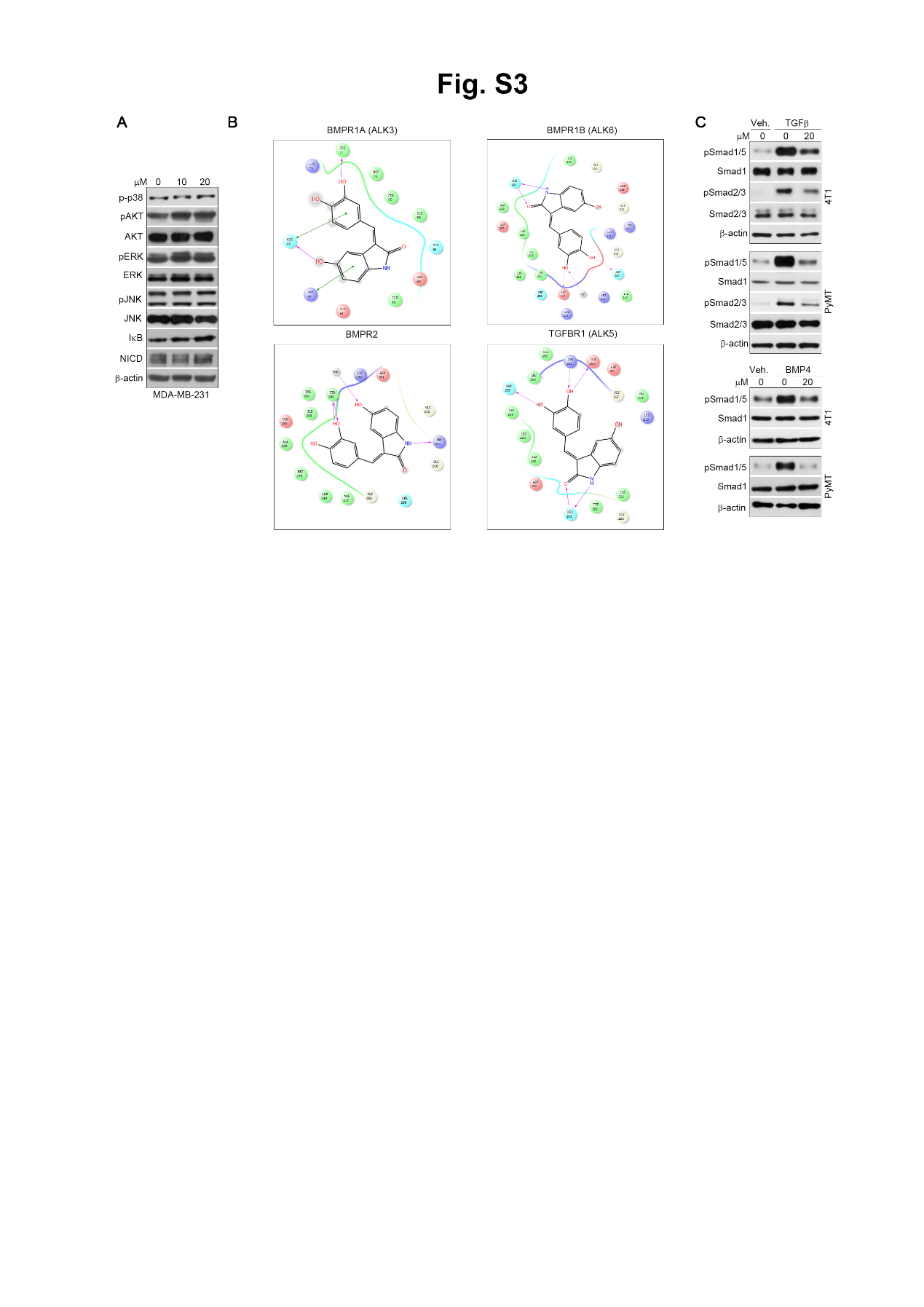

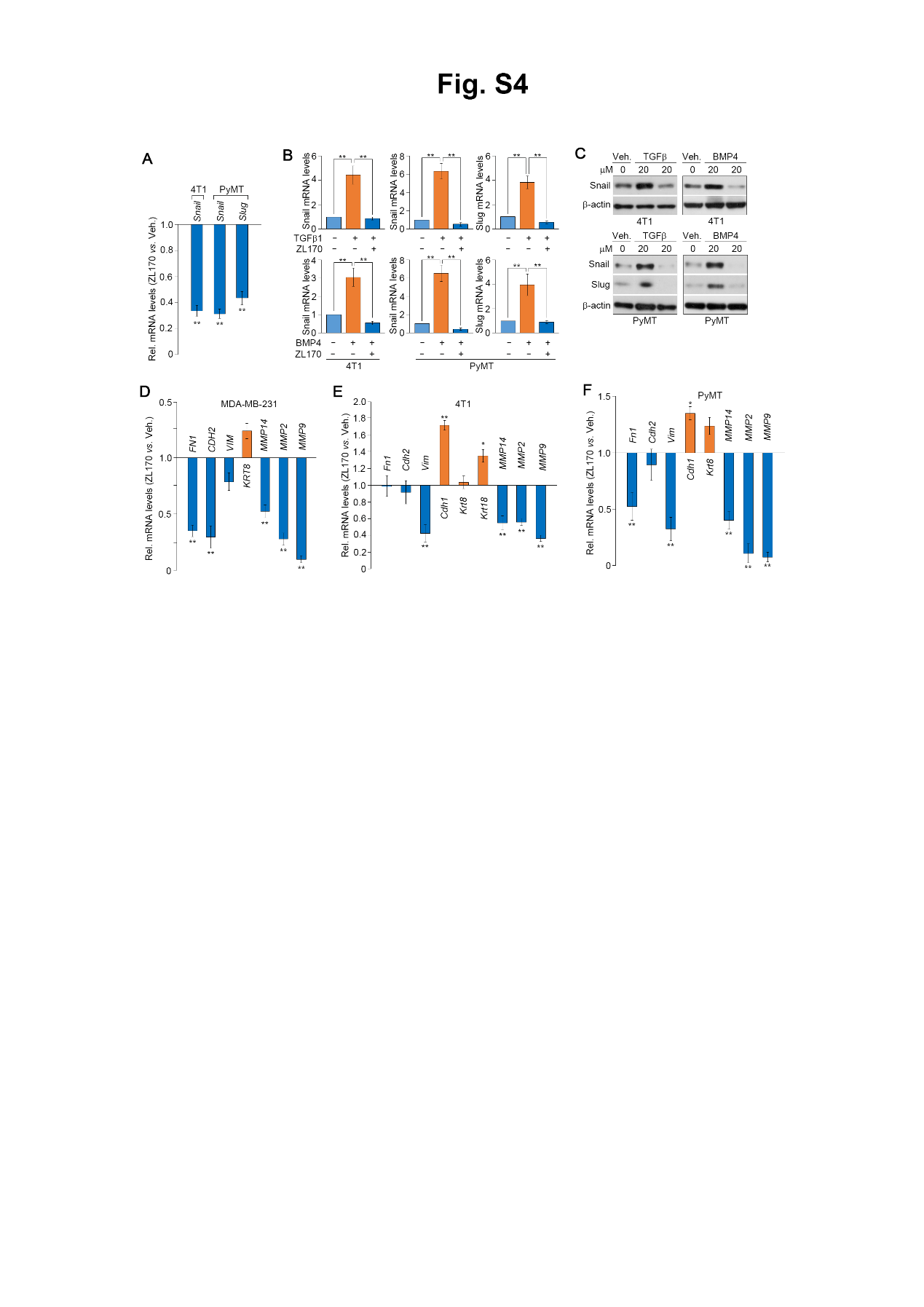

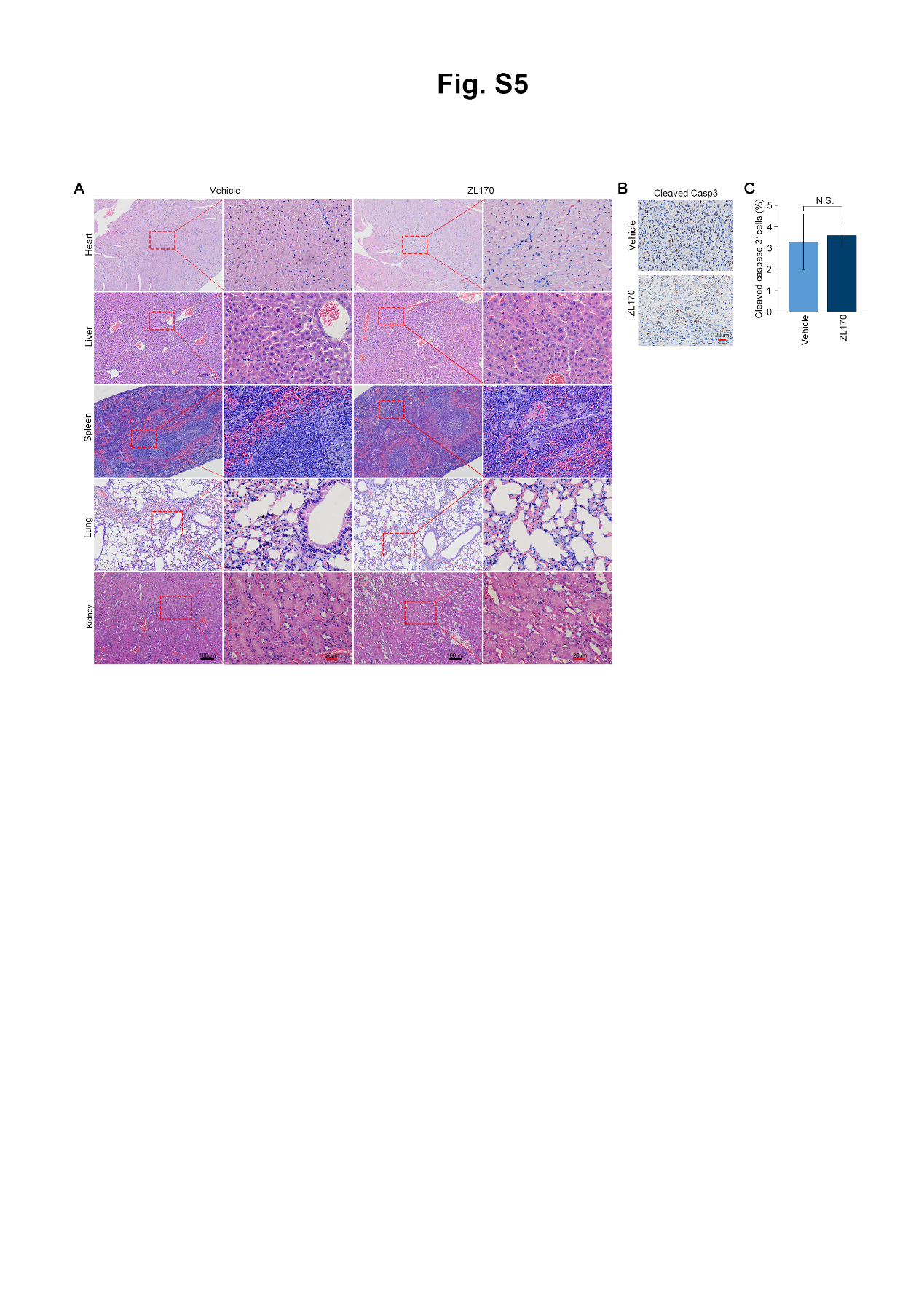

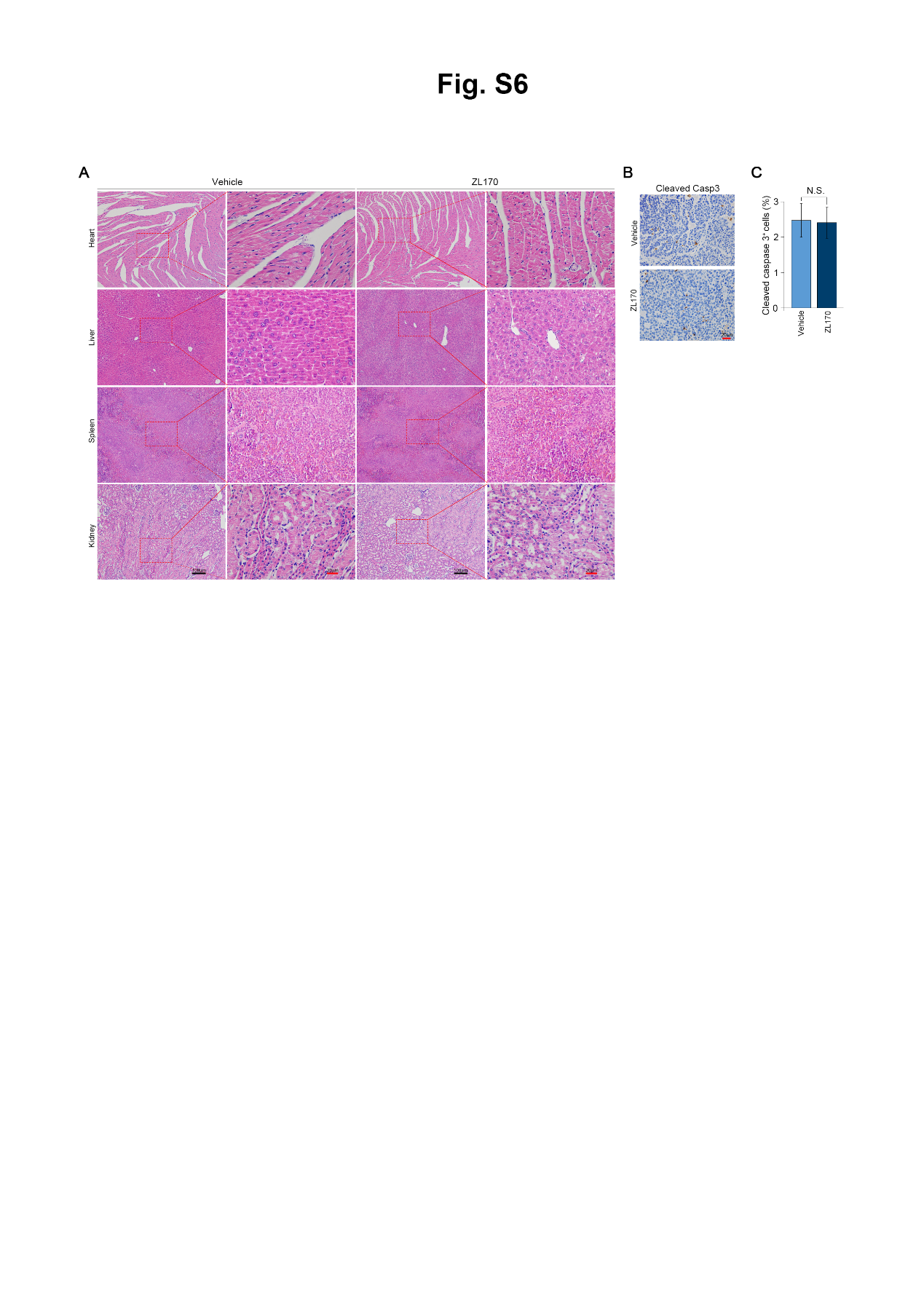

Supplement: Supplementary file 1 — Figure S1. Extraction, total synthesis and characterization of ZL170. Figure S2. ZL170 treatment does not induce C57BL6 mice death or any obvious adverse health effects. Figure S3. ZL170 is a potent dual inhibitor of TGFβ and BMP kinase receptors. Figure S4. ZL170 reverses the EMT program in TNBC cells. Figure S5. Administration of ZL170 does not induce apoptosis or elicit toxicity on key organs of tumor-bearing mice. Figure S6. ZL170 treatment does not induce apoptosis or elicit toxicity on key organs of PyMT transgenic mice. (DOCX 6822 kb) [file 13046_2019_1130_MOESM1_ESM.docx]
